# Hydroxytyrosol: lack of clastogenicity in a bone marrow chromosome aberration study in rats

**DOI:** 10.1186/1756-0500-7-923

**Published:** 2014-12-16

**Authors:** Laurie C Dolan, Hana Hofman-Hüther, Nicole Amann

**Affiliations:** Burdock Group, 859 Outer Road, Orlando, FL 32814 USA; Department of in vitro Pharmacology/Toxicology, BSL BIOSERVICE Scientific Laboratories GmbH, Behringstr. 6/8, 82152 Planegg, Munich, Germany; Wacker Chemie AG, Johannes-Hess-Str. 24, Burghausen, 84489 Germany

**Keywords:** Hydroxytyrosol, Rat, Chromosome aberration

## Abstract

**Background:**

Hydroxytyrosol is naturally found in olives, olive oil and wine, and is consumed as part of a normal diet. The substance may have utility as a preservative in a wide variety of foods due to its antioxidant, antimicrobial and amphipathic properties. The potential for hydroxytyrosol to cause chromosome aberrations *in vitro* had been tested previously, with positive results at high concentrations. An OECD Guideline 475 study (mammalian bone marrow chromosome aberration test) was conducted in rats with the oral limit dose of 2000 mg/kg bw to determine whether hydroxytyrosol is a clastogen *in vivo*.

**Results:**

The oral limit dose of 2000 mg/kg hydroxytyrosol was well tolerated by most rats; however, some rats exhibited clinical signs that abated within 24 hours. Treatment with hydroxytyrosol did not significantly enhance the number of aberrant cells or the mitotic index 24 or 48 hours post-dose. The positive control (cyclophosphamide) induced the expected increase in chromosomal aberrations and a decrease in the mitotic index, confirming the validity of the assay.

**Conclusion:**

An oral limit dose of 2000 mg/kg hydroxytyrosol does not induce chromosome aberrations in bone marrow cells of the rat. Accordingly, hydroxytyrosol is not a clastogen *in vivo*.

## Background

**Figure 1:**
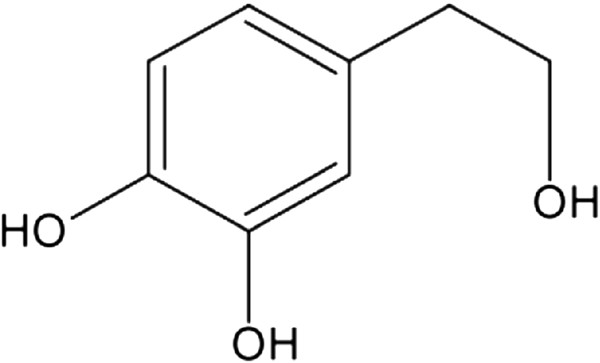
**Chemical structure of hydroxytyrosol.**

Hydroxytyrosol (Figure 1) is a phenolic substance present in olives, olive oil and wine in part *per* million (ppm) quantities [1, 2]. It greatly contributes to the shelf life of olive oil, preventing its oxidation [3]. Several studies have shown that hydroxytyrosol exhibits both antioxidant and antimicrobial properties [4–8], fueling interest in the substance as a food ingredient. Hydroxytyrosol could potentially be used as a preservative in a wide variety of foods, as it is soluble in both lipid and water fractions.

Genotoxicity testing is often required for new food ingredients. The ability of pure hydroxytyrosol to cause clastogenicity has been tested previously in an *in vitro* chromosome aberration test in human lymphocytes [9]. While there was no effect of the lowest concentration of hydroxytyrosol on the number aberrant cells (excluding gaps), the middle and high concentrations caused statistically significant increases in the number aberrant cells (excluding gaps) in the presence and/or absence of a metabolic activation system. The results of the study suggest that at high concentrations, hydroxytyrosol may cause clastogenicity. As recommended by the Organisation for Economic Co-operation and Development (OECD) and The European Food Safety Authority (EFSA), an *in vivo* study should be performed to assess whether the genotoxic potential observed *in vitro* is expressed *in vivo* [10, 11]. The purpose of the current study was to evaluate the clastogenic potential of hydroxytyrosol in rats according to the OECD 475 Guideline (mammalian bone marrow chromosome aberration test).

### Research hypothesis

We hypothesized that hydroxytyrosol was not clastogenic *in vivo* and performed a study in rats with an oral limit dose of 2000 mg/kg bw to test the hypothesis.

## Methods

The study was conducted at BSL BIOSERVICE Scientific Laboratories GmbH (BSL), Planegg/Munich, Germany. Healthy Wistar rats (7–13 weeks old obtained from Charles River (Sulzfeld, Germany)) were randomly distributed to five different treatment groups. Animals had free access to feed (Altromin 1324 maintenance diet) and tap water with the exception of an overnight fast (minimum 16 hours) before treatment. The facilities and procedures complied with the requirements of Commission Directive 2010/63/EU and the national legislation defined in the animal protection law (in the version of 22 September 2010) concerning the protection of animals used for experimental and other scientific procedures [12]. In accordance with European Animal Welfare Regulations, the protocol for the study was reviewed by members of the internal BSL ethics committee, submitted to local authorities and approved by the Government of Bavaria “Regierung von Oberbayern, Fachgebiet Tierversuche und Tierschutz (Az. 55.2.1.54-2532.0-11-14; Anzeige nach § 8a Abs. 1 TierSchG; Toxizität und Genotoxizität. Based on the literature data and animal welfare, a pre-experiment was regarded as not necessary and the study was performed as a limit dose study. The limit dose is utilized in situations when the test material is likely to be nontoxic, or having toxicity at levels above regulatory limit doses, suggested by the OECD for this study at 2000 mg/kg bw [10].

The test material was dissolved in distilled water one hour before treatment, and administered *via* gavage (10 ml/kg body weight (bw)) to two groups of five males and five females. The oral limit dose of 2000 mg/kg (bw) was evaluated. Two groups of five animals *per* sex (negative controls) were dosed with vehicle (distilled water) only. Five male and five female rats served as positive controls and received 40 mg/kg bw cyclophosphamide (CPA) in physiological saline by intraperitoneal injection. Body weights were measured prior to treatment to ensure that the weight of each animal was within 20% of the mean weight of each sex, as recommended by the guideline. All animals were examined for clinical signs of toxicity at various time points after treatment.

Four hours before scheduled euthanization (24 and 48 hour time points for both treated and negative control animals (5/sex/time-group) and 24 hours for the positive control group), the rats received 2 mg/kg colchicine (a metaphase arresting agent) by intraperitoneal injection. At termination, femurs were removed and bone marrow was harvested by cutting off the epiphyses and flushing the marrow out with a potassium chloride solution (0.4%). Collected cells were incubated (37°C for 25 min) and fixed with ten drops of ice-cold fixing solution (3:1 methanol: glacial acetic acid) under vigorous mixing. Cell suspensions were then spun in a centrifuge (200 x *g* for 10 min). The supernatant was discarded and the sediment containing the cells was resuspended in 4 ml of ice-cold fixing solution. The fixing procedure was repeated twice. Microscope slides were prepared by dropping the cell suspension on clean slides, flame-drying, and staining with Giemsa. All slides were independently coded (blinded) before microscopic examination using 100X oil immersion objectives.

At least 100 well-spread metaphases *per* animal were scored for cytogenetic damage (chromosome breaks, fragments, deletions, exchanges and disintegrations) unless a distinct positive result was observed in fewer than 100 metaphases [10]. Gaps and polyploidy were recorded but were not included in the calculation of the aberration rates. A minimum of 1000 cells *per* animal were analyzed for mitotic index (percentage of cells in mitosis), to determine the extent of bone marrow cell cytotoxicity. The assay was considered valid if (1) the weights of the rats did not exceed ± 20% of the mean weight of each sex, (2) the negative control vehicle did not result in a biologically significant increase in the number of cells with chromosomal aberrations compared to historical controls and (3) the CPA positive control produced a biologically significant increase in the number of cells with chromosomal aberrations. As described in the OECD 475 guideline, the test material would be considered positive for clastogenicity if there was a clear and dose-dependent increase in the number of cells with aberrations and a biologically relevant response (*i.e*., greater than the laboratory historical negative control range of 0.0% - 5.0% aberrant cells for males and 0.0% - 3.0% aberrant cells for females) for at least one dose group. A result was considered negative if there was no biologically relevant increase in the percentages of aberrant cells above concurrent controls, at any dose level.

The nonparametric Mann–Whitney test was performed to evaluate the 24 and 48 hour post-treatment aberrant cell (excluding gaps) and mitotic index data derived from the test. Mean values and standard deviations were calculated for the incidence of aberrant cells in each group. For the mitotic index, a mean value was determined *per* group. Differences from the corresponding negative control were considered to be statistically significant at the 95% confidence level (p < 0.05) for each variable examined. In accordance with the OECD 475 guideline, biological relevance was the primary criterion for interpreting the assay results, although statistical evaluation was permitted to aid in the interpretation. For this test biological relevance and statistical significance were considered together.

## Results

The mean body weights (± standard deviations) of the male and female rats were 224.9 ± 8.4 g and 155.1 ± 7.1 g, respectively. The body weight ranges for males (208–236 g) and females (139–165 g) were within ±6.2% of the mean for males and ±8.4% of the mean for females, well within the ±20% recommended by the guideline.

As shown in Table 1, after the administration of the 2000 mg/kg bw dose, 6/10 male rats showed a slight reduction in spontaneous activity within the first half hour. From the 1 hour time period onward, males did not exhibit this sign. The females did not show any signs of toxicity during the period of treatment, with the exception of one female rat that exhibited reduced spontaneous activity, ataxia, wasp waist, piloerection, and moving the bedding after 30 minutes. Some of these findings were still present in the female after 4 hours, but not after 24 hours. The results suggest that the oral limit dose of 2000 mg/kg caused some signs of acute toxicity in some, but not all animals.

**Table 1:** **Clinical signs of toxicity at the oral limit dose (2000 mg/kg bw)**

Observation	Time (post application)						
	30 min	1 hour	2 hours	3 hours	4 hours	24 hours	48 hours
Number of animals	10/sex	10/sex	10/sex	10/sex	10/sex	10/sex	10/sex
Reduced spontaneous activity	6 M, 1 F	0 M, 1 F	0 M, 1 F	0 M, 1 F	0 M, 1 F	0 M, 0 F	0 M, 0 F
Ataxia	0 M, 1 F	0 M, 1 F	0 M, 1 F	0 M, 1 F	0 M, 0 F	0 M, 0 F	0 M, 0 F
Wasp waist	0 M, 1 F	0 M, 1 F	0 M, 0 F	0 M, 0 F	0 M, 0 F	0 M, 0 F	0 M, 0 F
Piloerection	0 M, 1 F	0 M, 1 F	0 M, 1 F	0 M, 1 F	0 M, 1 F	0 M, 0 F	0 M, 0 F
Moving the bedding	0 M, 1 F	0 M, 1 F	0 M, 1 F	0 M, 0 F	0 M, 0 F	0 M, 0 F	0 M, 0 F
Salivation	0 M, 1 F	0 M, 0 F	0 M, 0 F	0 M, 0 F	0 M, 0 F	0 M, 0 F	0 M, 0 F
Prone position	0 M, 1 F	0 M, 0 F	0 M, 0 F	0 M, 0 F	0 M, 0 F	0 M, 0 F	0 M, 0 F

F = females; M = males.

Mean values of aberrant cells in the 24 and 48 hour negative control and test groups (Table 2) were not different from the concurrent negative control and were within the historical range for negative control values for the laboratory. Therefore, treatment with hydroxytyrosol did not significantly enhance the number of aberrant cells at the 24 or 48 hour post-dose time points (p > 0.05 for both). Interestingly, the incidence of aberrant cells in the male rats exposed to hydroxytyrosol for 24 hours was lower than the negative control value (p < 0.05). The mitotic index values for the 24 hour and 48 hour test groups were not different from the values for the corresponding negative controls, indicating that hydroxytyrosol was not toxic to the cells at the administered dose. The positive control induced the expected increase in chromosomal aberrations and a decrease in the mitotic index (p < 0.05). The validity of the assay was verified by (1) the weight variation of the rats not exceeding ± 20% of the mean weight of each sex, (2) the lack of biologically significant increases in aberrant cell values in the negative control group, and (3) the significant induction of aberrant cells in the CPA positive control group.

**Table 2:** **Summary of chromosome aberration assay results**

Study groups (***n*** = 5/sex)	Metaphases	Aberrant cells excluding gaps		Gaps	Polyploid cells	Mitotic index (% ± SD)
		**Number**	**(% ± SD)**			
Negative Control, 24 h						
Male	500	6	1.2 ± 1.1	5	0	11.3
Female	500	5	1.3 ± 1.4	8	0	8.4
Treatment Group, 24 h						
Male	500	0	0.0 ± 0.0*	4	0	11.0
Female	500	2	0.4 ± 0.9	7	0	10.1
Negative Control, 48 h						
Male	500	3	0.6 ± 1.6	4	4	10.7
Female	500	0	0.0 ± 0.0	3	0	11.4
Treatment Group, 48 h						
Male	500	1	0.2 ± 0.4	5	0	14.3
Female	500	1	0.2 ± 0.4	3	1	13.4
Positive Control, 24 h						
Male	250	100	40.0 ± 12.3*	6	1	3.1*
Female	250	108	43.2 ± 4.9*	11	0	2.5*

*Significantly different from negative control (distilled water) (p < 0.05).

n = number of animals; SD = standard deviation.

## Discussion

The results of the current study indicate that at an oral limit dose of 2000 mg/kg bw, hydroxytyrosol does not cause a statistically significant increase in the number aberrant cells (excluding gaps) in the bone marrow of rats. These results contradict results of a previous *in vitro* chromosome aberration test in human lymphocytes [9]. The latter study showed a statistically significant increase in the number of human lymphocytes with chromosomal aberrations (excluding gaps) when treated with 287.7 μg/mL hydroxytyrosol (1.9 mM) in the presence of S9 (3.5% in treated *vs*. 0.5% in control), or 503.5 μg/mL (3.3 mM) hydroxytyrosol in the absence (9.0% in treated *versus* 0.5% in control) or presence of S9 (4.5% in treated *vs*. 0.5% in control). There was no effect of 164.4 μg/mL (1.1 mM) hydroxytyrosol (the lowest dose tested) on the incidence of human lymphocytes with chromosomal aberrations in the absence or presence of S9. The test was considered valid, as the positive controls showed distinct increases in cells with chromosomal aberrations including or excluding gaps. The reason for the positive response *in vitro* is unknown, but may be related to the anti-oxidative nature of hydroxytyrosol. A number of antioxidants have been shown to exhibit pro-oxidative and DNA-damaging properties at high concentrations [13].

A possible reason for the negative result *in vivo* and the positive result *in vitro* is that the concentrations used in the *in vitro* study were higher than the concentration present in the serum of rats exposed to an oral limit dose of 2000 mg/kg bw. In rats provided 55 mg/kg bw hydroxytyrosol by the oral route, the maximal plasma concentration of hydroxytyrosol is 0.89-3.26 μg/ml (5.7 – 21 μM) [14]. Assuming absorption of hydroxytyrosol increases linearly with concentration, the maximal plasma concentration of hydroxytyrosol in rats exposed to the 2000 mg/kg limit dose in the current study may have reached 0.21 – 0.76 mM (32 – 115 μg/mL), which is lower, but not much different, than the lowest concentration of hydroxytyrosol used in the *in vitro* study (1.1 mM, also expressed as 164.4 μg/mL). Therefore, the lack of clastogenic effect at the lowest dose in the recent *in vitro* study [9] is in agreement with the results in the current *in vivo* study. There is also a low potential for cytotoxic actions in this or the *in vitro* study, as hydroxytyrosol has been found to inhibit hydrogen peroxide-induced cytotoxicity at 0.25 mM [15], a concentration similar to the calculated maximal plasma concentration in the current study. *In vitro*, hydroxytyrosol decreases cytotoxic actions in rat liver slices at a concentration of 50 μg/mL [16], within the range of the calculated rat plasma hydroxytyrosol levels determined in the current study. In healthy humans ingesting 25 ml virgin olive oil, the maximum plasma concentration of hydroxytyrosol is 26 μg/L (0.17 μM) [17], more than 6470 times lower than the lowest concentration of hydroxytyrosol used in the *in vitro* study. Because the positive results in the *in vitro* study were obtained with high concentrations of hydroxytyrosol that are not physiologically feasible (even in rats dosed with 2000 mg/kg bw hydroxytyrosol or a heavy consumer of olive oil), they are irrelevant for assessing risk of genotoxicity from consumption of hydroxytyrosol. The oral bioavailability of hydroxytyrosol when administered in olive oil or in an aqueous solution was found to be 99% and 75%, respectively, but is also rapidly eliminated in the urine due to preferential renal uptake of hydroxytyrosol [18]. The majority of hydroxytyrosol and its primary metabolites (sulfo-conjugated derivative, 3,4-dihydroxyphenylacetaldehyde and 4-hydroxy-3-methoxyphenylacetic acid) are removed from the body within the first 24 hours [18, 19].

## Conclusion

Based on the negative findings of the OECD Guideline 475 study with the limit dose in rats, one can conclude that there is no risk of clastogenicity of hydroxytyrosol, even in heavy consumers of olive oil or potential users of foods containing hydroxytyrosol as an added ingredient.

## Abbreviations

CPA: Cyclophosphamide
EFSA: The European Food Safety Authority
OECD: Organization for Economic Co-operation and Development.
